# Metalearning-Based Fault-Tolerant Control for Skid Steering Vehicles under Actuator Fault Conditions

**DOI:** 10.3390/s22030845

**Published:** 2022-01-22

**Authors:** Huatong Dai, Pengzhan Chen, Hui Yang

**Affiliations:** School of Electrical Engineering and Automation, East China Jiaotong University, Nanchang 330013, China; 2017029081100002@ecjtu.edu.cn (H.D.); pzchen@ecjtu.jx.cn (P.C.)

**Keywords:** fault-tolerant control, skid steering vehicle, reinforcement learning (RL), metalearning, torque distribution

## Abstract

Using reinforcement learning (RL) for torque distribution of skid steering vehicles has attracted increasing attention recently. Various RL-based torque distribution methods have been proposed to deal with this classical vehicle control problem, achieving a better performance than traditional control methods. However, most RL-based methods focus only on improving the performance of skid steering vehicles, while actuator faults that may lead to unsafe conditions or catastrophic events are frequently omitted in existing control schemes. This study proposes a meta-RL-based fault-tolerant control (FTC) method to improve the tracking performance of vehicles in the case of actuator faults. Based on meta deep deterministic policy gradient (meta-DDPG), the proposed FTC method has a representative gradient-based metalearning algorithm workflow, which includes an offline stage and an online stage. In the offline stage, an experience replay buffer with various actuator faults is constructed to provide data for training the metatraining model; then, the metatrained model is used to develop an online meta-RL update method to quickly adapt its control policy to actuator fault conditions. Simulations of four scenarios demonstrate that the proposed FTC method can achieve a high performance and adapt to actuator fault conditions stably.

## 1. Introduction

Due to their simple mechanical structure and flexible control, skid steering distributed drive vehicles are widely applied in various scenarios, including the construction industry, wheeled robots, agricultural vehicles, military vehicles, and so on. Generally, a skid steering vehicle has four independent driving wheels, forming a redundant actuator system, which delivers remarkable maneuverability and more options for control methods [1]. With the development of artificial intelligence (AI) technology, some recent research has applied the RL algorithm to the torque distribution of skid steering vehicles, providing new insights into vehicle control mechanisms. In real-world applications, more actuators in a system increases the probability of actuator faults. Therefore, a reasonable FTC method is very important for skid steering vehicles with redundant drive systems.

**Figure 1:**
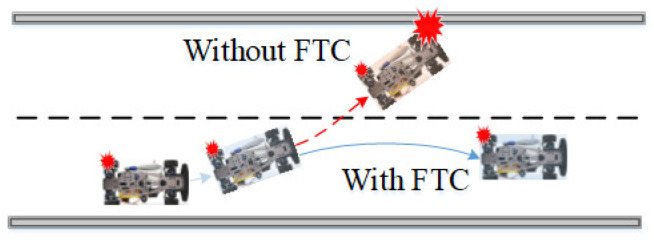
Scenarios of a skid steering vehicle’s fault. The red dashed line indicates the vehicle’s potential running path without FTC, which would cause a collision with nearby facilities. The blue line shows the expected vehicle driving path with FTC, which can avoid the collision risk.

The types of actuator faults in driving wheels are complex and may include additive faults, stuck-at-fixed-level faults, loss-of-effectiveness, and so on. In addition to the large number of possible faults, certain faults never seen before may occur during operation [2], causing vehicles to operate in an unstable state or even leading to catastrophic events. For example, assume a skid steering vehicle is operating on the road, as depicted in Figure 1, when an unexpected fault on the rear left wheel compromises the stability of the vehicle. Without FTC, the vehicle would operate under an instability condition and collide with nearby facilities; conversely, with FTC, the vehicle’s collision risk may be avoided [3].

To deal with such unforeseen and undesired faults, the controller must quickly learn their models under new runtime conditions and adapt proper torque distribution accordingly. A large variety of conventional methods are applied to guarantee the stability of the faulty vehicle in fault conditions, such as sliding mode control [4], robust control method [5], multi-agent control [6], and so on. These methods require however the explicit knowledge of specific failures and how these changes affect the system’s dynamical model in order to design resilient controllers. Traditional RL models can learn torque distribution policies based on feedback from the environment and have shown better performance than traditional methods [7]. However, the training mechanism of RL follows a trial-and-error manner; thus, the agent requires a large number of training episodes to learn an efficient strategy. The cost of computational resources and learning time is unacceptable when addressing the FTC problem with skid steering vehicles.

Metalearning is a recent method developed to “learn to learn” by leveraging optimization techniques [8]. Different from traditional RL, metalearning involves learning an initial parametrized control strategy from multiple relevant tasks, then relies on the obtained strategy to improve its performance on target tasks without training from scratch. Combining metalearning and RL, meta-RL has been widely studied for online FTC of systems [9]. This study, in combination with the DDPG-based torque distribution method, proposes a meta-DDPG-based FTC method for skid steering vehicles so as to improve the tracking performance when an actuator fault occurs. We first design an agent for torque distribution based on the DDPG algorithm; then, we construct an experience replay buffer with various actuator faults and train a metatrained model in the offline stage. Based on the metatrained model, the agent can quickly adapt to the faulty vehicle’s model through a small number of online iterations.

The main contributions of this study are as follows: (1) We develop a driving torque distribution method based on DDPG, which can perform dual-channel control over longitudinal speed and yaw rate. (2) We construct an offline actuator fault dataset—based on this, the meta-DDPG-based FTC method is proposed to quickly adapt to the vehicle’s model with actuator faults and to improve the desired value of tracking performance in the degraded conditions. To the best of our knowledge, this is the first work to deploy the meta-RL paradigm in the FTC of skid steering vehicles.

The remainder of this paper is as follows. Section 2 introduces related works concerning meta-RL and DDPG. The problem formulation is described in Section 3. Section 4 introduces the FTC method based on meta-DDPG, including the agent design of torque distribution and the meta-DDPG training approach. Section 5 presents the simulation environment and setting. We validate the proposed method with diverse simulation scenarios in Section 6. Conclusions are provided in Section 7.

## 2. Related Work

### 2.1. Traditional Control Methods for Skid Steering Vehicles

The traditional control methods for skid steering vehicles can be summarized into two categories: kinematic methods and dynamic methods. Kinematic methods are simple to implement but cannot effectively prevent the independently driving wheels from slipping. Kinematic methods in applications usually ignore wheel slip [10,11] or simply use the wheel speed following controller [12,13] to predict and compensate for such slippages.

Given that kinematic methods are not capable of overcoming wheel slippages, dynamic methods are proposed for this purpose to improve vehicle performance. Dynamic methods usually convert the vehicle control problem into an optimization problem of wheel torque distribution, then establish the objective function according to the optimization theory to solve the optimal solution of the system [14,15]. In [16], the directed yaw control method is investigated based on drivers’ operation intention, with an allocation control algorithm designed with a dynamic efficiency matrix for the stability control of electric vehicles. In [17], a coordinated, adaptive, robust control scheme integrated with a torque allocation technique is proposed to solve the chattering phenomenon and achieve high control performance under different ground conditions. Theoretically, dynamic methods are effective, and wheel slippage can be effectively controlled within the designed safety bound. However, their implementation requires accurate information on vehicle models and continuous estimation of terrain parameters, which are often influenced by complex and fluctuating wheel–ground interactions [18].

### 2.2. Meta-RL for Addressing System Failures and External Disturbances

Meta-RL aims to solve the new RL tasks by leveraging the experience learned from a set of similar tasks. Representatively, model-agnostic metalearning (MAML) [19] is purposed to optimize the initial parameters of the base learner in the metatraining process, which can significantly improve the efficiency of RL in the new task. The idea underlying meta-RL is that an internal representation exists that is broadly suitable for many tasks, such that slight tuning of the parameters can produce high-performance results on a new task. Meta-RL can easily adapt to related new tasks without a large amount of training, as is the case for classical RL. Many researchers have applied this feature to quickly adapt to system failures and external disturbances. In [20,21,22,23], the authors presented a series of methods based on meta-RL to quickly adapt their control policies to maintain degraded performance when faults occur in the aircraft fuel transfer system. The scheme of FTC methods includes offline metatraining and online metatesting stages. In [24], the authors presented a meta-RL-based approach to improve trajectory tracking performance for unmanned aerial vehicles (UAV) under actuator faults and disturbances. The proposed reference trajectory update method makes the system with an actuator fault follow the desired trajectory without needing access to the control inputs. They leveraged meta-RL to quickly adapt the system’s model at runtime using a small amount of online data for prediction and reference trajectory correction. In [25], an impact-angle guidance law based on meta-RL and the model predictive path integral (MPPI) was used for the interception of a maneuvering target using a varying velocity interceptor under partial actuator failures. The deep neural dynamic was used as a predictive model, and the control command was computed using MPPI. With the online adaption ability provided by metalearning, the deep neural dynamic can learn the changes and perturbations in the environment, and thus, has better tracking performance than the standard MPPI method. In [26], an adaptive controller based on meta-RL was proposed for automatic train velocity regulation to maintain the desired velocity. Due to complicated railway environments and the uncertain dynamics of the system, the velocity regulation problem is formulated as a sequence of stationary Markov decision processes (MDP) with unknown transition probabilities. This meta-RL algorithm learns the adaptive controller, which regulates the velocity of the train to a target value under changing operating conditions. In [27], the authors proposed the meta twin delayed deep deterministic policy gradient (meta-TD3) to realize the control of UAVs, allowing UAVs to quickly track a target for which the motion is uncertain. As meta-RL has shown great potential in quickly adapting to system failures and external disturbances in recent years, it provides new insights into the FTC of skid steering vehicles and is incorporated into our work.

### 2.3. DDPG for Complex System Control

In recent years, RL has been successfully implemented in complex control systems. In particular, the DDPG algorithm has a continuous action space and is very suitable for practical applications in the optimization of complex continuous action control processes [28,29]. In [30], the authors designed a real-time moving object tracking system with continuous actions for unmanned skid-steered vehicles (USSV) based on TD3. The TD3 algorithm with a soft updated replay buffer has high efficiency in the training process and high accuracy in the evaluation process. In [31], the DDPG algorithm was utilized to deal with the intelligent vehicle trajectory planning for continuous inputs and continuous outputs, which lessened the lateral control errors. In [32], the DDPG algorithm was adopted to optimize torque distribution control for a multiaxle electric vehicle with in-wheel motors. In [33], an end-to-end automatic lane changing method was proposed for autonomous vehicles using the DDPG algorithm. In [34], a Proportional–Integral–Derivative (PID)-Guide controller was designed to continuously learn through RL according to the feedback of environment to achieve high-precision attitude control of spacecraft. In [35], a controller based on the Robust-DDPG algorithm was developed for UAVs to fly stably in uncertain environments; the controller can continuously control two desired variables (roll and speed) of the UAV. In [36,37], the DDPG algorithm was used to automatically tune a torque-vectoring controller at a wide range of different vehicle velocities and under different friction surface conditions. The DDPG algorithm is suitable for system optimization of continuous actions in a dynamic environment and does not require an accurate model [38]. So, in this work, the driving torque distribution strategy of skid steering vehicles is based on the DDPG algorithm.

## 3. Problem Formulation: Meta-RL-Based FTC

In this work, we use MAML as the metalearning approach, which is one of the representative gradient-based meta-RL algorithms. We consider a metalearning model represented by a parameterized function $f_{\theta }$ with parameters θ. During metatraining, the model’s parameters θ are initialized randomly and updated to $\theta_{i}^{\prime }$ while adapting to fault $F_{i}$:

(1) $$\theta_{i}^{\prime }=\theta -\alpha \nabla_{\theta }L_{F_{i}}\left(f_{\theta }\right)$$

Metaoptimization is conducted across the faults; then, model parameters are updated according to Equation (2):

(2) $$\theta \leftarrow \theta -\beta \nabla_{\theta }\sum_{F_{i}∼p\left(F\right)}L_{F_{i}}\left(f_{\theta_{i}^{\prime }}\right),$$

where α and β are hyperparameters for optimization step size.

Our approach mirrors MAML, and the whole procedure of metalearning can be split as two steps: metatraining and metatesting. In MAML, the metatraining samples are randomly selected from a population $p_{i}∼P$ defining the MDP. These samples are applied to derive intermediate parameters. In this work, we forego the sampling process, but instead, exploit the experience with different actuator faults. In other words, MAML evaluates multiple processes on a single set of parameters. We propose to evaluate a single process on multiple sets of parameters.

**Figure 2:**
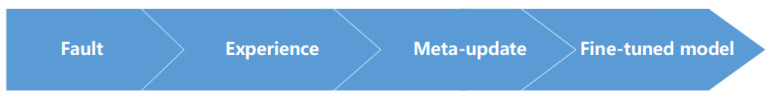
Flowchart of the FTC method.

The process of FTC is described as follows: FTC step begins with an abrupt fault, causing a discontinuous change in the process dynamics $p\to p^{*}$. In the aftermath of the fault, the agent continues to interact with $p^{*}$ and records the states, actions, and rewards in an online memory buffer $B_{online}$ by using its current policy parameters θ. Once sufficient interactions are buffered, the offline metatrained model is adapted, obtaining a fine-tuned model with updated parameters $\theta^{*}$. This fine-tuned model is then used to distribute the driving torque under the actuator fault condition. The proposed FTC method operates under the flowchart depicted in Figure 2.

## 4. The Meta-DDPG-Based FTC Method

This section will first briefly introduce the DDPG algorithm and design an agent for torque distribution for skid steering vehicles. Then, the meta-DDPG-based FTC method will be elaborated, which includes actuator fault dataset collection, metatraining, and the metatrained model update method.

### 4.1. The DDPG-Based Torque Distribution Method

The DDPG algorithm is adopted in this work to learn the control policy for torque distribution. As an Actor–Critic algorithm, the DDPG includes a Critic network and Actor network. The action-value neural network $Q\left(s,a|\theta^{Q}\right)$ is used in Critic to evaluate the value of taking an action *a* in state *s*, and the actor neural network $\mu \left(s|\theta^{\mu }\right)$ used in Actor is a function used to map a state *s* to a deterministic policy *a*, where $\theta^{Q}$ and $\theta^{\mu }$ are the network parameters. The actor policy $\mu \left(s|\theta^{\mu }\right)$ is updated as follows:

(3) $$\nabla_{\theta_{\mu }}{\mu |}_{s_{t}}\approx \frac{1}{N}\sum_{t}\left(\nabla_{a}Q\left(s,a|\theta^{Q}\right){|}_{s=s_{t},a=\mu \left(s_{t}\right)}\right.\left.\nabla_{\theta_{\mu }}\mu \left(s|\theta^{\mu }\right){|}_{s=s_{t}}\right).$$

For learning the *Q*-value, Bellman’s principle of optimality is used to minimize the root mean squared loss:

(4) $$y_{t}=r\left(s_{t},a_{t}\right)+\gamma Q^{\prime }\left(s_{t+1},\mu^{\prime }\left(s_{t+1}|\theta^{\mu^{\prime }}\right)|\theta^{Q^{\prime }}\right)$$

(5) $$L=\frac{1}{N}*\sum_{t}{\left(y_{i}-Q\left(s_{i},a_{i}|\theta^{Q}\right)\right)}^{2}.$$

Equation (4) is used to calculate the reward value and Equation (5) shows the calculation formula of the loss function. The DDPG is a model-free, off-policy algorithm, and the design of the agent’s reward function, state space, and action space have an important impact on the control performance.

#### 4.1.1. State Space

**Figure 3:**
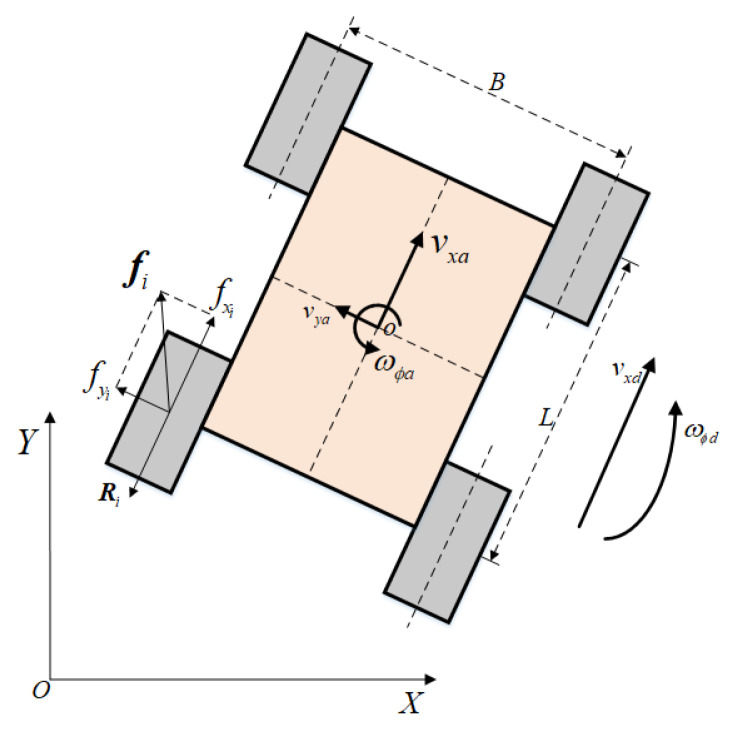
Skid steering vehicle diagram.

The control objective is the actual longitudinal speed and yaw rate, used to track the desired value effectively and accurately. The state space includes the error of longitudinal speed $v_{delta}$ and yaw rate $\omega_{delta}$, which are defined as follows:

(6) $$\begin{array}{cc} v_{delta} & =v_{xd}-v_{xa}\\ \omega_{delta} & =\omega_{\phi d}-\omega_{\phi a}, \end{array}$$

where $v_{xd}$ and $\omega_{\phi d}$ are the desired value of the longitudinal speed and the yaw rate, respectively; $v_{xa}$ and $\omega_{\phi a}$ are the corresponding actual value, respectively. The desired value and actual value are shown in Figure 3. The longitudinal acceleration $v_{xa}^{\prime }$ and the angular acceleration $\omega_{\phi a}^{\prime }$ are also used as variables of the state space, which seriously affect the vehicle’s maneuverability. The state space is defined as follows:

(7) $$state=\left\{v_{delta},\;\omega_{delta},\;v_{xa}^{\prime },\;\omega_{\phi a}^{\prime }\right\}.$$

#### 4.1.2. Action Space

Vehicles’ behavior depends on their driving torque on wheels, geometry, and the ground. It is assumed in this study that the geometry and the ground do not have any change. Therefore, the driving torque to each wheel is used as the action space variables. Thus, the action space is defined as follows:

(8) $$action=\left\{T_{fl},\;T_{rl},\;T_{fr},\;T_{rr}\right\}.$$

#### 4.1.3. Reward Function

The reward function acts as a signal to evaluate the performance when taking an action *a* at a state *s*. The rewards are the only feedback signals available for the agent’s learning. Reasonably designing the reward function is the key to guide the agent to obtain an effective control strategy. Design of the reward function is mainly based on the vehicle’s maneuverability, which is reflected in reducing errors related to longitudinal speeds and yaw rates. Therefore, to ensure the performance of vehicles, a well-defined reward function provided at every time step is introduced:

(9) $$\begin{array}{cc} R= & -\left(v_{delta}^{2}+5\times \omega_{delta}^{2}\right)-0.01\times \left({\left(v_{xa}^{\prime }\right)}^{2}+{\left(\omega_{\phi a}^{\prime }\right)}^{2}\right)\\ \; & -0.001\times \left(T_{fl}^{2}+T_{rl}^{2}+T_{fr}^{2}+T_{rr}^{2}\right)+D. \end{array}$$

The first term in the reward function above encourages the agent to minimize errors with longitudinal speeds and yaw rates. The second and third terms in the reward function encourage the agent to reduce the action value when the error is within a certain range, to prevent the error from not converging. The last term is a large positive reward when the agent is close to the ideal conditions and is defined as below:

(10) $$D=\left\{\begin{array}{cc} 3000, & \mathrm{if}\;\left|v_{delta}\right|<0.01\times \left|v_{xd}\right|\;\mathrm{and}\;\left|\omega_{delta}\right|<0.01\times \left|\omega_{\phi d}\right|\\ 0, & \mathrm{otherwise}. \end{array}\right.$$

In this way, a large positive reward is applied when the agent is close to the ideal conditions.

### 4.2. Meta-DDPG-Based FTC Framework

**Figure 4:**
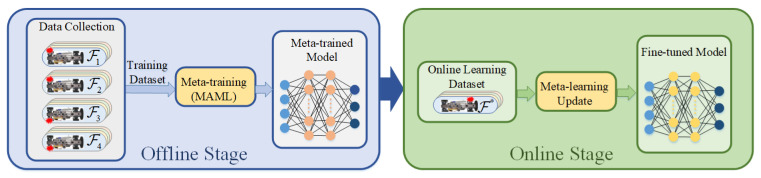
Framework of the meta-DDPG-based FTC.

The proposed FTC method follows the traditional gradient-based meta-RL framework—MAML—which consists of offline and online stages. The traditional design of MAML mainly focuses on evaluating multiple processes on a single set of parameters. However, the proposed FTC method aims to evaluate multiple sets of parameters corresponding to different faults in one process. A dataset with various actuator faults is established during the offline stage, and a metatrained model is obtained through metatraining with this dataset. During the online stage, the adaptation step begins with an abrupt actuator fault, which could be a different fault than the ones used in the offline stage. Sufficient interactions are collected in the aftermath of the fault; then, the metatrained model is fine-tuned with the online data. Finally, a fine-tuned model is obtained and applied to distribute the driving torque under the actuator fault condition. The framework of the meta-DDPG-based FTC is demonstrated in Figure 4.

#### 4.2.1. Actuator Fault Dataset

The actuator faults of vehicles are complex, and novel faults may still occur during operations. Although the traditional RL adopts exploration to ensure the policy has a certain generalizability, the previous policy cannot be used directly when dealing with different faults [39]. Aiming to resolve the problem of insufficient generalizability for different faults in the implementation process of RL, the meta-RL optimizes the initial parameters of RL by randomly sampling and training different faults in the metatraining stage.

In this work, vehicles with various actuator faults are tasked to tracking different desired values. For each actuator fault $F_{i}\left(i=1,2,\cdots ,n\right)$, a fault experience replay buffer $B_{F_{i}}$ is constructed; the experience $\langle s_{t}^{F_{i}},a_{t}^{F_{i}},r_{t}^{F_{i}},s_{t+1}^{F_{i}}\rangle$, generated by the agent interacting with the environment, is stored in the corresponding $B_{F_{i}}$; finally, all experience replay buffers are integrated to form the offline dataset $B_{offline}$. The dataset $B_{offline}$ contains the data from each fault; $F_{i}\in F$ also includes the cases of vehicles without actuator faults tracking different desired values. During metatraining, an equal number of experiences are randomly selected from each fault experience replay buffer for training to realize the learning of different faults.

#### 4.2.2. Metatraining of Meta-DDPG

Metatraining of the meta-DDPG is a process to learn the faults dataset $B_{offline}$ and obtain a metatrained model, purposed to maximize the expected generalization ability of the RL algorithm in all trained actuator faults. When responding to a new fault, based on the previous initial parameters learned through metatraining, the metatrained model would enable the agent to use only a few data points and training iterations to adapt the vehicle’s model with actuator fault.

Metatraining mainly includes two update processes: internal RL for a single task and external metalearning update for multiple different tasks. In meta-DDPG, updates of internal DDPG training and external metalearning are performed alternately by meeting a certain update frequency. DDPG learns multiple faults separately to obtain different parameters, and meta-DDPG obtains the initial parameters of RL by optimizing different parameters.

The function $f_{\theta }$ with parameters θ is a strategy for mapping a state *s* to an action *a*. When a new fault $F_{i}$ occurs, the strategy function adapts to the new fault, and the parameter θ becomes $\theta_{i}^{\prime }$ through the one-step gradient descent.

(11) $$\theta_{i}^{\prime }=\theta -\alpha \nabla_{\theta }L_{F_{i}}\left(f_{\theta }\right),$$

where α is the step size. The initial parameters θ is obtained by calculating the average loss as follows:

(12) $$\min_{\theta }\sum_{F_{i}∼p\left(F\right)}L_{F_{i}}\left(f_{\theta_{i}^{\prime }}\right)=\min_{\theta }\sum_{F_{i}∼p\left(F\right)}L_{F_{i}}\left(f_{\theta }-\alpha \nabla_{\theta }L_{F_{i}}\left(f_{\theta }\right)\right).$$

Metaoptimization is used to optimize the initial policy parameters such that only a few stochastic gradient descents will produce a maximally effective strategy on the new task. The metaoptimization formula is as follows:

(13) $$\theta \leftarrow \theta -\beta \nabla_{\theta }\sum_{F_{i}∼p\left(F\right)}L_{F_{i}}\left(f_{\theta_{i}^{\prime }}\right),$$

where β is the meta step size, and $L_{F_{i}}$ is the loss function, which corresponds to the reward function *R* in the RL. The reward function *R* is described in Section 4.1.3. The loss for fault $F_{i}$ and model $f_{\theta }$ takes the following form:

(14) $$L_{F_{i}}\left(f_{\theta }\right)=-E_{s,a∼f_{\theta }}\left[\sum_{t=1}R_{i}\left(s_{t},a_{t}\right).\right]$$

The whole algorithm for the metatraining process of the FTC method is described in Algorithm 1.
**Algorithm 1** metatraining of meta-DDPG algorithm1:Randomly initialize critic network and actor network with weights $\theta^{Q}$ and $\theta^{\mu }$;2:**for**meta_iteration=1,2,⋯,M**do**3:    Initialize a random fault $F_{i}∼p\left(F\right)$;4:    **for** t=1,2,⋯,N **do**5:        Sample trajectories from $F_{i}$ using policy $f_{\theta }$;6:        Calculate the metalearning loss function of fault $F_{i}$:                                $L_{F_{i}}\left(f_{\theta }\right)=-E_{s,a∼f_{\theta }}\left[\sum_{t=1}R_{i}\left(s_{t},a_{t}\right)\right]$;7:        Update the adapted parameters with gradient descent: $\theta_{i}^{{}^{\prime }}=\theta -\alpha \nabla_{\theta }L_{F_{i}}\left(f_{\theta }\right)$;8:        Sample trajectories by using adapted policy $f_{\theta_{i}^{\prime }}$ in $F_{i}$;9:      **end for**10:    Metaupdate $\theta \leftarrow \theta -\beta \nabla_{\theta }\sum_{F_{i}∼p\left(F\right)}L_{F_{i}}\left(f_{\theta_{i}^{\prime }}\right)$;11:**end for**

#### 4.2.3. Update of the Metatrained Model

In the metatraining process, the metatrained model is learned with a well-generalized initialization of parameters θ. By applying the metatrained model to the nominal vehicle model, we can quickly obtain the torque distribution controller of the vehicle without actuator fault through the conventional DDPG training process.

At runtime, as the vehicle experiences a fault $F^{*}$, the agent collects *K* consecutive data by using its current policy and constructs an online learning dataset in a memory buffer $B_{online}$. With this runtime dataset, the offline metatrained model is adapted according to Algorithm 2, and the fine-tuned policy $f_{\theta^{*}}$ with updated parameters $\theta^{*}$ is obtained. The agent is able to quickly collect enough data to easily adapt its model, because the model initial parameters are optimized by metatraining in the offline stage.
**Algorithm 2** Update of the metatrained model1:**for**episode=1,2,⋯,K**do**2:    $\theta^{*}\leftarrow \theta$;3:    **for** t=1,2,⋯,N **do**4:        Sample trajectories from $F^{*}$ using policy $f_{\theta }$;5:        Calculate the metalearning loss function of fault $F^{*}$:                                $L_{F^{*}}\left(f_{\theta }\right)=-E_{s,a∼f_{\theta }}\left[\sum_{t=1}R_{i}\left(s_{t},a_{t}\right)\right]$;6:        Update adapted parameters with gradient descent: $\theta^{*}\leftarrow \theta^{*}-\alpha \nabla_{\theta }L_{F^{*}}\left(f_{\theta }\right)$;7:    **end for**8:**end for**

## 5. Simulation Environment and the Training Result

### 5.1. Control Model Used during Simulations

In this work, we establish a dynamic model for the skid steering vehicle with four independent driving wheels [40]. Figure 3 shows that the friction between the wheels and ground result in the vehicle’s motion. Thus, with Newton’s Second Law, the dynamic model of the vehicle system can be described as:

(15) $$\begin{array}{cc} m\left(v_{xa}^{\prime }-v_{ya}\omega_{\phi a}\right) & =\sum_{i=1}^{i=4}f_{x_{i}}-\sum_{i=1}^{i=4}R_{x_{i}}+d_{x}\\ m\left(v_{ya}^{\prime }+v_{xa}\omega_{\phi a}\right) & =\sum_{i=1}^{i=4}f_{y_{i}}+d_{y}\\ J\omega_{\phi a}^{\prime } & =\sum_{i=1}^{i=4}\tau_{f_{i}}+\sum_{i=1}^{i=4}\tau_{R_{i}}+d_{\phi }, \end{array}$$

where *m* is the mass of the vehicle and *J* is its moment of inertia around the center of gravity (CG); $\tau_{f_{i}}$ and $\tau_{R_{i}}$ denote the torque applied to the vehicle around the CG by the tractive force $f_{i}=f_{x_{i}}\hat{x}+f_{y_{i}}\hat{y}$ and the rolling resistance force $R_{i}$ at the *i*th wheel, respectively; $\hat{x}$ and $\hat{y}$ indicate the unit vectors in longitudinal and lateral directions, respectively; $d_{x}$, $d_{y}$, and $d_{\phi }$ represent disturbances; $R_{i}$ is the rolling resistance force shown in Equation (16).

(16) $$R_{i}=-\mu N_{i}\hat{x},$$

where μ is the coefficient of the rolling resistance, which is related to the type of ground and the wheel characteristics; $N_{i}$ is the normal force at the *i*th wheel, which is associated with the mass of the vehicle and its CG position. The second term of Equation (15) represents lateral motion. To simplify the problem, this study does not consider the lateral motion of the vehicle. The specifications of the dynamic model are enumerated in Table 1.

The effect of actuator faults on the vehicle systems is demonstrated by the actuator fault modeling. The most common faults of the in-wheel motors are considered, including additive fault, stuck-at-fixed-level fault, and loss-of-effectiveness [41]. The generalized form of actuator faults is defined as follows:

(17) $$T_{ij}=\varepsilon_{ij}T_{d\_ij}+\Delta T_{ij}=\left\{\begin{array}{c} \varepsilon_{ij}=1,\&\Delta T_{ij}=0\left(Healthy\right)\\ \varepsilon_{ij}=1,\&\Delta T_{ij}=const\left(Additive\;fault\right)\\ \varepsilon_{ij}=0,\&\Delta T_{ij}=const\left(Stuck\;fault\right)\\ \varepsilon_{ij}\in \left(0,1\right),\&\Delta T_{ij}=0\left(Loss\;of\;effectiveness\right), \end{array}\right.$$

where $T_{ij}$ is the actual wheel torque, $\varepsilon_{ij}$ denotes the loss-of-effectiveness gain, $T_{d\_ij}$ is the desired wheel torque, and $\Delta T_{ij}$ represents the extra torque caused by a fault, which is assumed to be bounded. It is assumed in this study that the actuator fault information can be obtained in the fault diagnosis and detection methods [42].

This study simulates actuator faults by assigning different model parameters to different wheels. During the offline stage, metatraining data are collected with a nominal vehicle model and with vehicle models under four different actuator faults given in Table 2. At runtime, the vehicle with testing faults is tasked with tracking different desired values. Two testing faults are listed in Table 3, which include the front left wheel fault that appeared within the training bounds $\varepsilon_{fl}=0.2$ of the commanded torque on the front left wheel, and the rear right wheel fault outside of the training bounds—where the commanded torque on the rear right wheel adds a fixed offset $\Delta T_{rr}=20$ N·m.

### 5.2. Meta-DDPG Hyperparameter Settings

The simulation environment in this study was set up in Python 3.7 on PyCharm IDE. All training processes were implemented on an Intel Core i5 computer. The deep learning framework TensorFlow-2.0.0 was used to build the networks on a macOS system.

According to the definitions of the state space and the definition of the action space, the meta-DDPG model has a 4-dimensional input and a 4-dimensional output. In meta-DDPG, the policy neural networks are constructed as 4×512×512×4 and the Q-value neural networks are constructed as 8×512×512×1. The specific parameters of the meta-DDPG algorithm are listed in Table 4.

### 5.3. Training Results

**Figure 5:**
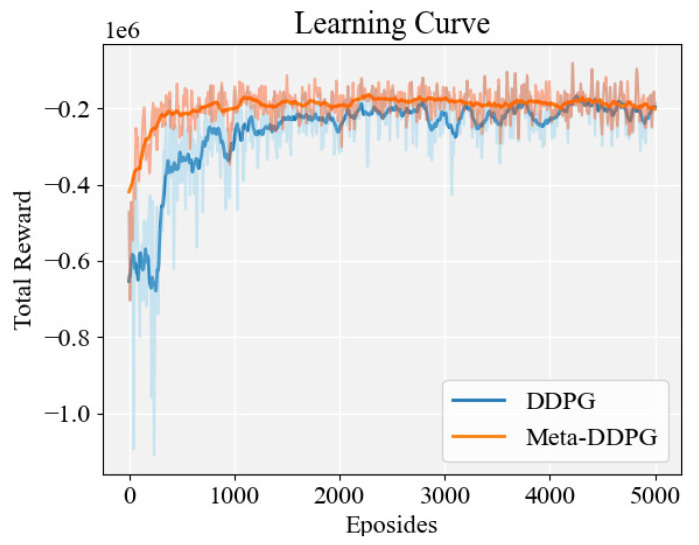
The total rewards’ trend in the metatesting.

After the metatraining stage, the obtained metatrained model had been trained 5000 times with the nominal vehicle model, and the torque distribution controller under the condition of no actuator fault was obtained. For comparison, with the same configuration, a DDPG-based torque distribution controller was also trained. The total rewards for meta-DDPG and DDPG are shown in Figure 5. The meta-DDPG can converge faster and fluctuates less than the DDPG-based model, obtaining a higher total reward. This shows that both controllers can be successfully used to distribute the driving torque of the skid steering vehicle; however, the meta-DDPG-based controller has a better control performance than the DDPG-based controller.

## 6. Results

In this section, we simulate three different conditions and compare the simulation results. The conditions are as follows: (1) no fault; (2) fault without FTC; (3) fault with the proposed FTC method. The simulation is conducted in two scenarios: straight scenario and constant steering scenario. In each scenario, the actuator faults are assigned as in Table 3, and the vehicle behaviors under the corresponding faults are discussed. The study cases are listed in Table 5.

As a commonly used evaluation method, the vehicles’ tracking performance are evaluated for their stability. Two functions are utilized to evaluate the longitudinal speed tracking performance and yaw rate tracking performance of vehicles. The integrals of the quadratic function of the deviations of both the longitudinal speed and the yaw rate from the desired value are used to evaluate the vehicle tracking control performance. The two integrals are denoted as $J_{1}$ and $J_{2}$, respectively [43].

(18) $$\begin{array}{c} J_{1}=\int_{0}^{t}v_{delta}^{2}\;d\tau \\ J_{2}=\int_{0}^{t}\omega_{delta}^{2}\;d\tau . \end{array}$$

### 6.1. Simulations in the Straight Scenario

**Figure 6:**
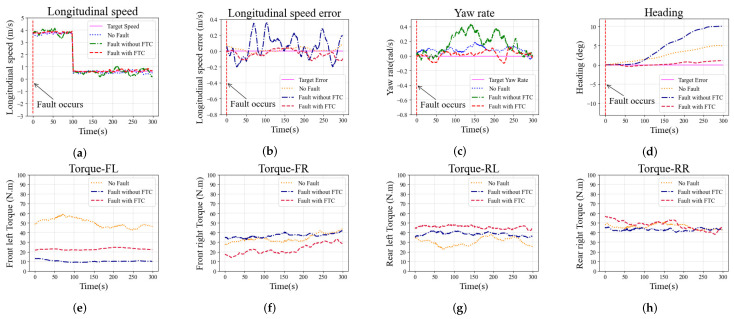
Simulation results in the straight scenario with fault $F_{1}^{*}$ on the front left wheel: (**a**) Longitudinal speed; (**b**) Longitudinal speed error; (**c**) Yaw rate; (**d**) Heading; (**e**–**h**) Torque on each wheel (front left, front right, rear left, and rear right).

**Figure 7:**
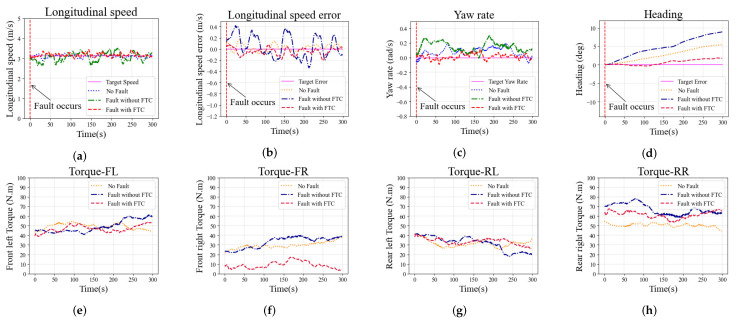
Simulation results in the straight scenario with fault $F_{2}^{*}$ on the rear right wheel: (**a**) Longitudinal speed; (**b**) Longitudinal speed error; (**c**) Yaw rate; (**d**) Heading; (**e**–**h**) Torque on each wheel (front left, front right, rear left, and rear right).

Simulations in the straight scenario are performed with a constant zero steering angle, so as to identify the drifts of the faulty vehicle. Two different actuator faults, $F_{1}^{*}$ and $F_{2}^{*}$, are employed in the simulations; the simulation results in the straight scenario are shown in Figure 6 and Figure 7, respectively.

The longitudinal speed is set to 3.8 m/s at the beginning, and 0.7 m/s at 100 s, while the yaw rate is set to 0 rad/s and kept constant. The longitudinal speed at different moments in the simulation is shown in Figure 6a. As observed in Figure 6b, fluctuations in the longitudinal speed in the case without FTC are significantly greater than in other cases. The errors with the longitudinal speed in cases with FTC and with no-fault demonstrate no significant difference, and the results in both cases are smaller than in the case without FTC. The yaw rates in different cases are shown in Figure 6c. The desired yaw rate is a constant zero in the straight scenario. The cases with FTC and no-fault show that errors with the yaw rate can be kept within 0.2 rad/s. However, without FTC, the errors exceeded 0.2 rad/s. As shown in Figure 6d, the heading of the vehicle without FTC has a larger errors than in other cases, indicating that the vehicle suffers a greater drift. The driving torques on each wheel are shown in Figure 6e–h. The case with FTC generates an additional torque on the rear left wheel to compensate for the front left wheel fault, while the torque on the front right wheel is reduced to balance the yaw of the vehicle. The above analysis shows that the vehicle’s tracking performance in the case of FTC is not much different from that in the case of no-fault, and it is significantly better than in the case without FTC, indicating that the proposed FTC method can improve the tracking performance of the vehicle when it encounters a fault within the training bounds.

The second simulation in the straight scenario is conducted with fault $F_{2}^{*}$ on the rear right wheel. The additive fault of the driving wheel is outside the offline training bounds, which is used to simulate the vehicle encountering a novel fault. Simulation results are shown in Figure 7 and have the same trend as the case with fault $F_{1}^{*}$. The longitudinal speed of the case without FTC fluctuates greatly, and the maximum error exceeds 0.4 m/s, as shown in Figure 7a,b. The longitudinal speed tracking performance shows no significant difference between the cases with FTC and no-fault, and the results of both of the cases are better than in the cases without FTC. The yaw rate tracking performance also shows the same trend, as shown in Figure 7c. The yaw rate error in the cases with FTC and no-fault is kept within 0.2 rad/s; without FTC, the error exceeds 0.2 rad/s. As shown in Figure 7d, the heading of the vehicle without FTC shows a larger error than in other cases, indicating that the vehicle has a greater drift. The driving torques on each wheel are shown in Figure 7e–h. The torque on the front right wheel is reduced to compensate for the rear right wheel’s additive fault. The above analysis is sufficient to demonstrate that the proposed FTC method can also improve the stability of vehicles when they encounter a fault outside of the training bounds.

**Figure 8:**
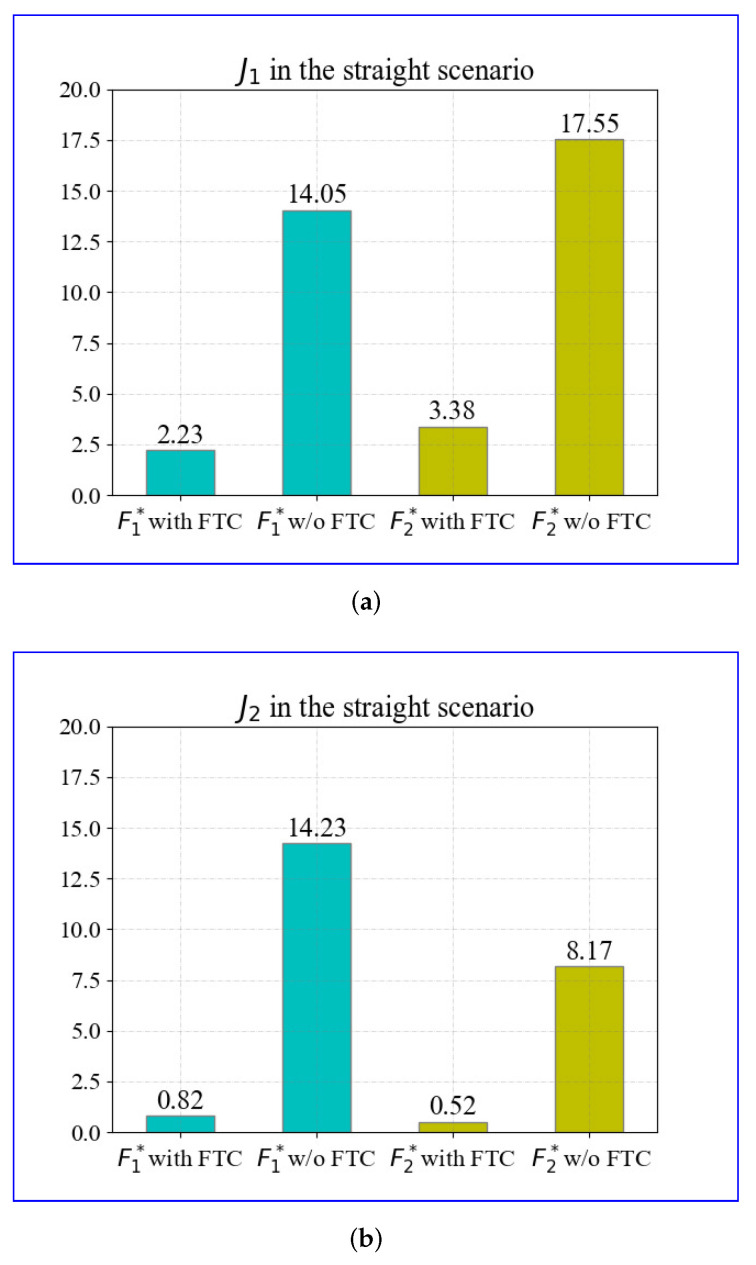
The evaluation in the straight scenario with FTC algorithm and without FTC algorithm: (**a**) $J_{1}$ in the straight scenario; (**b**) $J_{2}$ in straight scenario.

To quantitatively illustrate the improvement of the proposed FTC control strategy more specifically, Equation (18) is used to evaluate the performance of the longitudinal speed tracking and yaw rate tracking. The results of $J_{1}$ and $J_{2}$ in the straight scenario are displayed in Figure 8.

The evaluation results are obtained based on the aforementioned simulations. Comparisons of $J_{1}$ in cases with and without the FTC under 300 s of driving are displayed in Figure 8a. The FTC method reduces $J_{1}$ of the fault $F_{1}^{*}$ by 84.13% and the fault $F_{2}^{*}$ by 80.74%. The results of $J_{2}$ are shown in Figure 8b; the FTC method reduces $J_{2}$ of the fault $F_{1}^{*}$ by 94.23% and the fault $F_{2}^{*}$ by 93.64%. The evaluation results in the straight scenario demonstrate that the proposed FTC can effectively reduce the severity of actuator faults, including those outside of the training bounds, that is, those that had never occurred before.

### 6.2. Simulations in the Constant Steering Scenario

**Figure 9:**
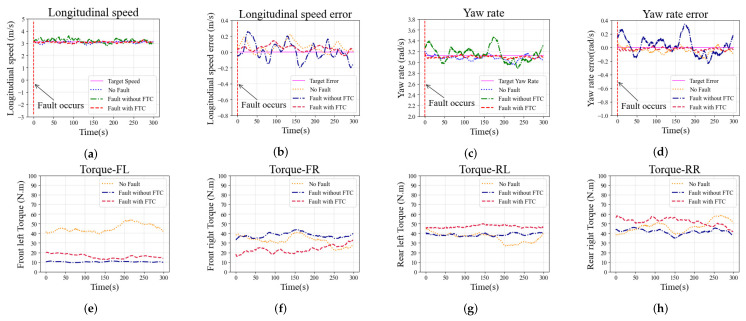
Simulation results in the constant steering scenario with fault $F_{1}^{*}$ on the front left wheel: (**a**) Longitudinal speed; (**b**) Longitudinal speed error; (**c**) Yaw rate; (**d**) Yaw rate error; (**e**–**h**) Torque on each wheel (front left, front right, rear left, and rear right).

**Figure 10:**
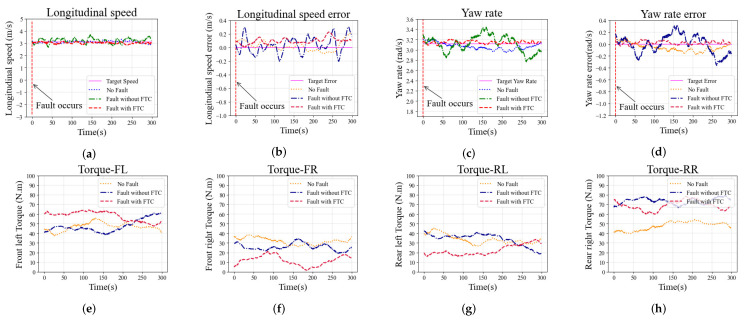
Simulation results in the constant steering scenario with fault $F_{2}^{*}$ on the rear right wheel: (**a**) Longitudinal speed; (**b**) Longitudinal speed error; (**c**) Yaw rate; (**d**) Yaw rate error; (**e**–**h**) Torque on each wheel (front left, front right, rear left, and rear right).

The second simulation study is conducted with constant steering of the vehicle to identify the vehicle’s cornering ability under actuator fault conditions. The simulation is performed at a constant 3.2 m/s longitudinal speed and a constant 3.0 rad/s yaw rate with two different fault conditions, and the fault types are consistent with those discussed in Section 6.1. The simulation results of constant steering with the faults $F_{1}^{*}$ and $F_{2}^{*}$ are shown in Figure 9 and Figure 10, respectively.

The longitudinal speed tracking and longitudinal speed error of the vehicle with fault $F_{1}^{*}$ during the simulation are shown in Figure 9a,b, respectively. The maximum longitudinal speed error of the case without FTC exceeds 0.2 m/s, the longitudinal speed fluctuates sharply, and the longitudinal speed tracking performance is worse than in the other two cases. For the cases with FTC and with no-fault, the longitudinal speed tracking error performance is not remarkably different, and both are better than in the case without FTC. The yaw rate tracking and the yaw rate error are shown in Figure 9c,d, respectively. The case with FTC shows that the yaw rate error is close to zero. However, without the FTC algorithm, the yaw rate fluctuates sharply and the yaw rate tracking error increases significantly. The driving torque on each wheel are shown in Figure 9e,h. Due to the fault $F_{1}^{*}$, the torque on the faulty front left wheel is significantly reduced. The case with FTC algorithm generates additional torque on the rear left wheel to compensate for the fault $F_{1}^{*}$, and the front right torque is reduced to balance the yaw of the vehicle. The comparison of torque distribution shows that the proposed FTC method can adapt to the impact of fault $F_{1}^{*}$ on the vehicle.

The same trend is observed in the case with fault $F_{2}^{*}$ in the constant steering scenario, as shown in Figure 10. The longitudinal speed and yaw rate fluctuations in the case without FTC are more severe than in the other two cases, and the tracking error performance is worse as well. Compared with the no-fault case, the longitudinal speed and yaw rate tracking performance of the case with FTC is similar or even better. Driving wheel torques with fault $F_{2}^{*}$ in the constant steering scenario are shown in Figure 10e–h. The torque on the rear right wheel with fault $F_{2}^{*}$ is significantly increased due to additive fault, and the front right torque is reduced to balance the yaw of the vehicle.

**Figure 11:**
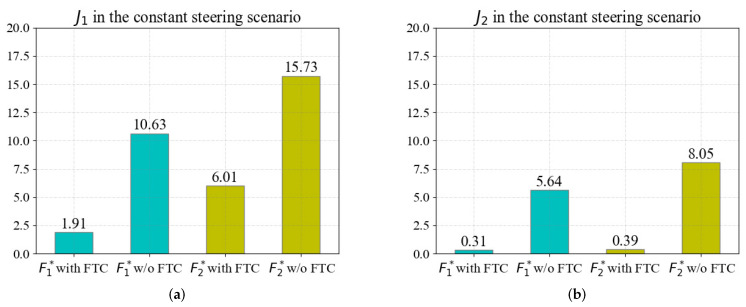
The evaluation in the constant steering scenario with FTC algorithm and without FTC algorithm: (**a**) $J_{1}$ in the constant steering scenario; (**b**) $J_{2}$ in the constant steering scenario.

The quantitative evaluation results in the constant steering scenario are shown in Figure 11, which are similar to those in the straight scenario. The results of $J_{1}$ are shown in Figure 11a; the FTC method reduces $J_{1}$ of the fault $F_{1}^{*}$ by 82.03% and the fault $F_{2}^{*}$ by 61.79%. As shown in Figure 11b, the FTC method reduces $J_{2}$ of the fault $F_{1}^{*}$ by 94.50% and the fault $F_{2}^{*}$ by 95.16%. The evaluation results demonstrate that the proposed FTC can effectively reduce the impact of actuator faults in the constant steering scenario, indicating that the proposed FTC method can effectively improve the tracking performance of the vehicle with faults in the constant steering scenario.

Based on all the simulation results illustrated above, the effect of the meta-DDPG-based FTC method on the skid steering vehicles’ longitudinal speed and yaw rate tracking have been investigated. In such vehicles, a single wheel fault can impose a serious impact on longitudinal speed and yaw rate tracking. However, such an impact can be overcome with the implementation of the FTC method proposed in this study.

## 7. Conclusions

In this work, we propose a meta-DDPG-based FTC method for skid steering vehicles moving under actuator faults. We have leveraged metalearning, which can quickly adapt the vehicle’s model at runtime using a small number of online data to maintain tracking performance. Based on the DDPG algorithm, we developed an agent that can perform dual-channel control over the longitudinal speed and yaw rate of skid steering vehicles. Considering the diversity of actuator faults, we designed the method to learn more general tracking policy by metalearning from a variety of actuator faults. We constructed an experience replay buffer with various actuator faults to provide data for multi-faults learning of RL algorithm. A metalearned model is trained from the data provided by the experience replay buffer in the offline stage. Based on the meta-learned model, the agent can quickly online adapt to the vehicle’s model with an actuator fault using a few gradient steps. Four types of testing scenarios were explored. The simulation results demonstrate that actuator faults can have a serious impact on tracking performance and the proposed FTC can effectively overcome this impact.

This work creates an exciting path towards using metalearning approaches for the FTC method of skid steering vehicles. A redundant driving system can provide a hardware platform to address the actuator FTC problems, making the novel FTC algorithm possible. Currently, we are exploring ways to extend this work by incorporating fault detection techniques. Time delays are critical in systems such as skid steering vehicles, and the study of actuator delays in vehicles will also be considered in future work. We also plan to apply the proposed method to different vehicle systems, such as independent driving electric vehicles as they are also prone to actuator faults.

## Figures and Tables

**Table 1:** Specifications of the dynamic model.

Description	Symbol	Value
Vehicle mass	*m*	2360 kg
Vehicle wheelbase	*B*	2.4 m
Vehicle length	*L*	5.0 m
Wheel radius	*r*	0.2 m
Yaw moment of inertia	*J*	4050 kg · m^2^
Rolling resistance coefficient	μ	0.05

**Table 2:** Fault types used during training.

Training Fault	Fault Type	Description
$F_{1}$	$\varepsilon_{rl}=0\;\&\;\Delta T_{rl}=20$	Stuck fault on rear left wheel
$F_{2}$	$\varepsilon_{rl}=0.2\;\&\;\Delta T_{rl}=0$	Loss-of-effectiveness on rear left wheel
$F_{3}$	$\varepsilon_{rr}=0\;\&\;\Delta T_{rr}=20$	Stuck fault on rear right wheel
$F_{4}$	$\varepsilon_{rr}=0.2\;\&\;\Delta T_{rr}=0$	Loss-of-effectiveness on rear right wheel

**Table 3:** Fault types used during testing.

Training Fault	Fault Type	Description
$F_{1}^{*}$	$\varepsilon_{fl}=0.2\;\&\;\Delta T_{fl}=0$	Loss-of-effectiveness on front left wheel
$F_{2}^{*}$	$\varepsilon_{rr}=1\;\&\;\Delta T_{rr}=20$	Additive fault on rear right wheel

**Table 4:** Parameters of model training.

Parameter Name	Parameter Value
Meta iteration	150
Metatesting max episodes	5000
Max episode steps	100
Memory capacity	1,000,000
Batch size	512
Actor-network learning rate	0.0001
Critic-network learning rate	0.001
Online dataset capacity	200

**Table 5:** Study cases.

	Straight Scenario		Constant Steering Scenario	
Fault	$F_{1}^{*}$	$F_{2}^{*}$	$F_{1}^{*}$	$F_{2}^{*}$
**Case**	Case 1	Case 2	Case 3	Case 4

## Data Availability

Not applicable.
